# Dissolved Gases Forecasting Based on Wavelet Least Squares Support Vector Regression and Imperialist Competition Algorithm for Assessing Incipient Faults of Transformer Polymer Insulation

**DOI:** 10.3390/polym11010085

**Published:** 2019-01-08

**Authors:** Jiefeng Liu, Hanbo Zheng, Yiyi Zhang, Xin Li, Jiake Fang, Yang Liu, Changyi Liao, Yuquan Li, Junhui Zhao

**Affiliations:** 1 Guangxi Key Laboratory of Power System Optimization and Energy Technology, Guangxi University, Nanning 530004, Guangxi, China; liujiefeng9999@163.com (J.L.); yiyizhang@gxu.edu.cn (Y.Z.); daisy_lixin@sina.com (X.L.); fangjiake123@gmail.com (J.F.); ly20111006@hotmail.com (Y.L.); gxchangyiliao@163.com (C.L.); 2 State Grid Henan Electric Power Research Institute, Zhengzhou 450052, Henan, China; liyuquancat_xjtu@163.com; 3 National Demonstration Center for Experimental Electrical Engineering Education, Guangxi University, Nanning 530004, Guangxi, China; 4 Department of Electrical and Computer Engineering & Computer Science, University of New Haven, West Haven, CT 06516, USA; JZhao@newhaven.edu

**Keywords:** transformer polymer insulation, dissolved gases, wavelet technique, imperialist competition algorithm, least squares support vector machine

## Abstract

A solution for forecasting the dissolved gases in oil-immersed transformers has been proposed based on the wavelet technique and least squares support vector machine. In order to optimize the hyper-parameters of the constructed wavelet LS-SVM regression, the imperialist competition algorithm was then applied. In this study, the assessment of prediction performance is based on the squared correlation coefficient and mean absolute percentage error methods. According to the proposed method, this novel procedure was applied to a simulated case and the experimental results show that the dissolved gas contents could be accurately predicted using this method. Besides, the proposed approach was compared to other prediction methods such as the back propagation neural network, the radial basis function neural network, and generalized regression neural network. By comparison, it was inferred that this method is more effective than previous forecasting methods.

## 1. Introduction

It is generally accepted that high-quality electrical energy is at the heart of the smart grid business [1,2,3,4], and the power transformer is one of the keys to guaranteeing that the business is reliable and successful. Transformers are widely distributed in power transmission and distribution systems. Hence, the failure of power transformers is often followed by disastrous consequences, which include equipment burning and large-scale blackouts. Therefore, the reliable and stable operation of power transformer is very necessary. In order to avoid blackouts, power companies use many methods for online monitoring of transformers. Some new methods including detecting hot spot temperature, windings displacement and dissolved gas analysis (DGA), have been used to detect incipient faults in the power transformer [5]. However, dissolved gas analysis (DGA) is one of the most widely used methods [6,7,8,9]. In an oil-immersed transformer, insulating oil and oil-immersed paper are extremely important factors in the insulation ability of power transformers. The heat generated by the normal operation of the transformer cannot break the oil-hydrocarbon molecular chemical bonds of the transformer. However, when partial overheating or arc high temperature occurs, most of the heat generated by the fault will act on the insulation oil and solid insulation. The insulation material will age and generate gas at the same time. Generally, carbon monoxide (CO), carbon dioxide (CO_2_) hydrogen (H_2_), acetylene (C_2_H_2_), ethylene (C_2_H_4_), ethane (C_2_H_6_), and methane (CH_4_) are considered to be the seven key gases. When the transformer is overheated or discharged, it will aggravate the gas production. According to thermal degradation principles, the content of key gases is directly related to the specific fault type. A number of simple schemes such as Doernerburg, Rogers and IEC, have been employed to provide effective diagnosis by using the contents of gas compositions. The early faults in the oil-immersed transformer and their development trend can be quickly identified if the content of the gas components is predicted based on the historical data of the transformer [10,11,12].

Recently, artificial intelligence (AI) has been widely researched for its application in the field of fault analysis [13,14,15,16,17,18,19,20,21,22,23,24]. For example, some AI methods such as support vector machines (SVM), artificial neural networks and fuzzy logic inference systems have been applied to diagnose the faults in transformers. Besides, some other machine learning algorithms such as support vector regression (SVR) have been utilized to forecast the operational conditions of equipment in the future. Compared to these methods, the application of SVM in abnormal detection and fault diagnosis has marked advantages. It overcomes the local minimum, dimension, and over-fitting problems, and requires less in the scale of the training sample. As a reformulation of the standard SVM [19], the least squares support vector machine (LS-SVM) was proposed by Suykens et al. [25] Compared with the traditional quadratic programming method, applying the linear least squares criterion to the loss function can better simplify the standard SVM. Taking the simplicity and inherited advantages of SVM, including the basic principle of structural risk minimization and kernel mapping, LS-SVM can be extended to pattern recognition and regression problems [26,27,28,29,30]. The wavelet function is orthonormal in *L*_2_ (*R*^N^) space [31,32,33], however, Gaussian and polynomial kernels commonly used for SVM are not orthonormal bases. So, the wavelet function can approximate arbitrary curves in *L*_2_ (*R*^N^) space. Therefore, the simulated results show that the approximation effect of the wavelet kernel is obviously better than that of the Gaussian kernel [32,33].

In order to build wavelet LS-SVM regression (W-LSSVR), wavelet technique and LS = SVM regression were combined in this paper. By using the global optimizer imperialist competition algorithm (ICA) [34], the regularization term of W-LSSVR and the hyperparameters in kernel function were optimized by cross-validation to minimize the error objective function. These two measure criteria, squared correlation coefficient (*r*^2^) and mean absolute percentage error (MAPE) guide the performance evaluation of the proposed W-LSSVR, and represent the learning and generalization abilities of the SVM estimator [35,36]. By analyzing and comparing the experimental results, satisfactory prediction accuracy and valuable information were obtained, which highlights the significance and novelty of the proposed method.

## 2. Wavelet Least Squares Support Vector Machine

The nonlinear capacity of LS-SVM is tied to use of the kernel trick in pattern recognition and regression analysis, which results in mapping the input by an optimal choice of kernel function to a higher dimensional feature space. In this study, the three kinds of wavelet kernels (Morlet, Marr and DOG wavelet kernels) [6] were employed.

As for problems of regression, the model is first taken into account in the original space of the formula below:

(1) $$f(x)=\omega^{T}\varphi (x)+\beta$$

where *x* ∈ *R^n^*, *f*(*x*) ∈ *R*, while *φ*(*x*) denotes a set of non-linear transformations. This leaves a training set {(*x*_1_, *y*_1_), …, (*x_l_*, *y_l_*)} ⊂ *R^n^* × *R*, where *x_i_* is the input value and *y_i_* is the correlative objective value for sample *i*. The target is to conclude an assessment *f*(*x*) that approximates the actual desired *y* from the acceptable training samples and is as planus as possible. In the raw space, the following optimization formulation is utilized to show the LS-SVM regression:

(2) $$\min\Phi (\omega ,e)=\frac{1}{2}\omega^{T}\omega +\frac{1}{2}C\sum_{i=1}^{l}e_{i}^{2}$$

Subject to the equality constrains:

(3) $$y_{i}=\omega^{T}\varphi (x_{i})+\beta +e_{i},i=1,2,\ldots ,l$$

Annotate *ω* might become potentially infinite dimensional, therefore, one cannot solve the primal optimization problem of this article that we have mentioned. Consequently, the above-mentioned optimization problem’s reformulation through a Lagrange functional into a dual optimization problem results in a method that is a function of the data, which is expressed in the primary dimensional feature space and the relevant formula is as below:

(4) $$f(x)=\sum_{i=1}^{l}\alpha_{i}K(x,x_{i})+\beta$$

## 3. Using the Imperialist Competition Algorithm to Optimize Hyper-Parameters

### 3.1. The Imperialist Competition Algorithm

Hyper-parameters play important roles in the W-LSSVR model, thus, the selection of the hyper-parameters will affect the performance of the W-LSSVR. The main objective is to find a better way to select the optimal choices from several candidate parameters by applying cross validation. Based on this idea, it is necessary to use more rigorous approaches such as analytical techniques and heuristic algorithms to get the best hyper-parameters. While gradient-based algorithms determine the hyper-parameters of analytical techniques, modern evolutionary algorithms like simulated annealing algorithms, the genetic algorithm and imperialist competition algorithm determine the hyper-parameters of heuristic algorithms [37,38,39]. In this paper, the W-LSSVR model is optimized by using the ICA [40].

To begin with, the ICA is used to initialize the population. Then, the scope needs to be searched using some specific procedures. Eventually, this algorithm ends with the optimal solution or near optimal solution. It is noted that initial population consisted of countries—imperialists refer to the most powerful countries, colonies are the other countries. The empire is formed by both imperialists and colonies. In ICA, the initial countries are represented by the array, so the array dimension *N* means the number of countries *N*, which is defined as [*p*_1_, *p*_2_, …, *p_n_*]. The cost of the *i*-th country *S_i_* can be calculated by Equation (5):

(5) $$S_{i}=F\left(country_{i}\right)=F\left(P_{i1},P_{i2},\cdots P_{iN}\right)$$

Some powerful countries (the countries with minimum cost) and *N_imp_* countries are selected to be the imperialists from the *N* initial countries. At the same time, the rest of the countries are colonies belonging to the imperialists. The ICA process aims to use the minimum cost to find the most powerful country.

### 3.2. Hyper-Parameter Optimization

When optimizing the hyper-parameter with ICA, each particle stands for a potential solution, which includes the kernel parameter *a* and regularization parameter *C*. The fitness function that is relative to the optimization problem being considered is applied to measure the hyper-parameter optimality. The goal of training and testing W-LSSVR is to minimize the errors between the actual values and the forecasting values of the testing samples, which can enhance the generalization performance of the regression model. Thus, the fitness function is shown as follows:

(6) $$Fitness=\frac{1}{k}\sum_{i=1}^{k}\sqrt{\frac{1}{m}\sum_{j=1}^{m}{\left(f(x_{ij})-y_{ij}\right)}^{2}}$$

where *k* is the number of folds in cross validation, *m* is the number of each subset as validation, *y_ij_* is the true value, and *f*(*x_ij_*) is the forecasting value of the validation samples.

According to the goal of minimizing the fitness function, the particle with the minimal fitness value should be reserved during the optimization process since it outperforms the other particles. Consequently, it is able to choose the optimal hyper-parameters.

The process of ICA for hyper-parameter optimization is presented in the following steps:

*Step 1*: Initialize parameters of ICA and set up fitness function model.

*Step2*: Divide all countries into two kinds, imperialists and colonies according to their costs. The countries with higher cost are empires while the countries with lower costs are colonies.

*Step 3*: If the colonial costs are lower than the imperial costs, exchange the empire and colony, if not, loop to the next step.

*Step4*: Use a differential evolution arithmetic operator and calculate the total cost of the empires.

*Step5*: Implement the competition of the imperialists.

*Step 6*: If there is an empire without any colonies, disintegrate the empires and preserve elite individuals.

*Step7*: When the selected condition is satisfied, the flow meets the termination condition. In this case, end the algorithm and get the optimal parameters. Repeat this process from step 1 if it does not meet the termination condition. The termination condition is defined below:

(1) The location of the united empire is the expected solution to the optimal problem because the unique empire controls all empires and colonies.
(2) Reaching the maximum generations.

**Figure 1:**
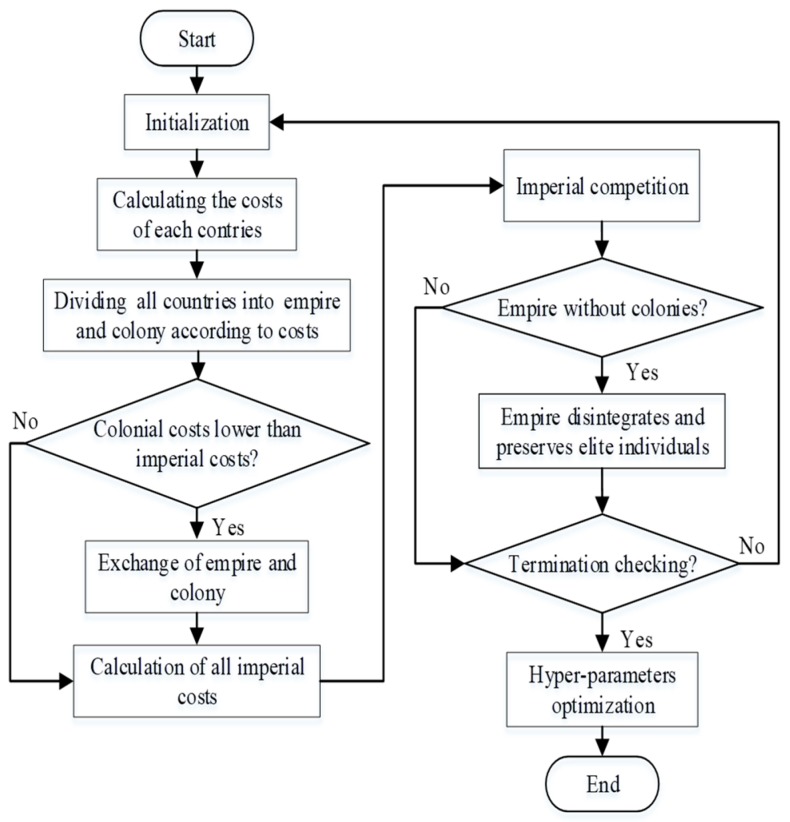
Flowchart of hyper-parameter optimization by using ICA.

Figure 1 shows the flowchart of ICA for hyper-parameter optimization:

## 4. Procedure for Forecasting Key Gas Contents with Wavelet Least Squares Support Vector Machine Regression and Imperialist Competition Algorithm

The various stages, which are based on the above-mentioned W-LSSVR and ICA processes are explained as follows. All the wavelet techniques and LS-SVM algorithms in this study were coded in MATLAB.

Stage 1: Data preprocessing

A collection of original data can be obtained from the crucial gas contents. After extracting the raw data, the training and testing sets can be generated separately. Since it is only possible to get the raw sample data from the power company on an irregular basis, the primary sampling data needs to be changed into an equal interval time series by means of interpolation methods—as can be seen, the Hermite spline interpolation [41] is used in this study. Finally, the raw data is normalized, which includes training and testing data, and which enhances the generalization ability of W-LSSVR.

Stage 2: Implement ICA to optimize hyper-parameters

In the optimizing process, cross validation is applied to ICA. The *k*-fold cross validation divides the training data into *k* disjunct sets when the training data is once substituted randomly. In the *i*-th (*i* = 1, 2, …, *k*) iteration, the performance of the model trained on the other *k* − 1 set (called training set) can be estimated by the *i*-th set (called the validation set). In the end, the mean value is used to get *k* different performance estimates.

Stage 3: Training and testing

With the optimal hyper-parameters obtained from ICA implementation, the W-LSSVR training model based upon the training data can be built, and the outputs based on the testing data will be forecasted.

It is possible to establish the W-LSSVR training model with the training data by using the optimal hyper-parameters determined from ICA implementation. Then, the output of the testing data is predicted.

In order to verify the performance in the training stages, the squared correlation coefficient (called *r*^2^) and mean absolute percentage error (MAPE) are used as evaluation indicators. However, in the testing stage, only MAPE is used as the evaluation indictor. Assuming *x*_1_, …, *x_l_* are the training data and *f*(*x*_1_), …, *f*(*x_l_*) are the predicting values by W-LSSVR. In addition, assuming *y*_1_, …, *y_l_*, are the true values, then, the *r*^2^ and MAPE can be defined as follows:

(7) $$r^{2}=\frac{{\left(l\sum_{i=1}^{l}f(x_{i})y_{i}-\sum_{i=1}^{l}f(x_{i})\sum_{i=1}^{l}y_{i}\right)}^{2}}{\left(l\sum_{i=1}^{l}f{\left(x_{i}\right)}^{2}-{\left(\sum_{i=1}^{l}f(x_{i})\right)}^{2}\right)\left(l\sum_{i=1}^{l}y_{i}{}^{2}-{\left(\sum_{i=1}^{l}y_{i}\right)}^{2}\right)}$$

(8) $$\mathrm{MAPE}=\frac{1}{l}\sum_{i=1}^{l}\left|\frac{f(x_{i})-y_{i}}{y_{i}}\right|$$

## 5. Results and Comparisons

### 5.1. Experimental Results Based upon Wavelet Least Squares Support Vector Machine Regression and the Imperialist Competition Algorithm

The dissolved gas data collected from several Chinese power companies in [6] are used as the key gas content data for oil-immersed transformers (H_2_, CH_4_, C_2_H_2_, C_2_H_4_ and C_2_H_6_) to demonstrate the effectiveness of the proposed forecasting model. The rating of the tested transformers is 110 kV.

In the study, the aforementioned three kinds of wavelet kernels including Morlet, Marr and DOG are investigated. For Case 1, the periodical sampling time was the period between November 2009 and January 2010. First, the experimental data, including training data and testing data were normalized before applying W-LSSVR. Then, taking the Morlet W-LSSVR as an example, the algorithm of ICA with mutation is implanted to find the optimal hyper-parameters for each group of the key gas contents by using 5-fold cross validation. The parameters of the ICA algorithm used in this paper are as follows: the number of countries and the number of initial imperialists were fixed to 20 and 6, respectively; the dimension of the optimized function was set to 2; the maximum number of generations was 100; the revolution rate was set to 0.3; the assimilation coefficient equaled 2, and the assimilation angle coefficient equaled 0.5.

**Figure 2:**
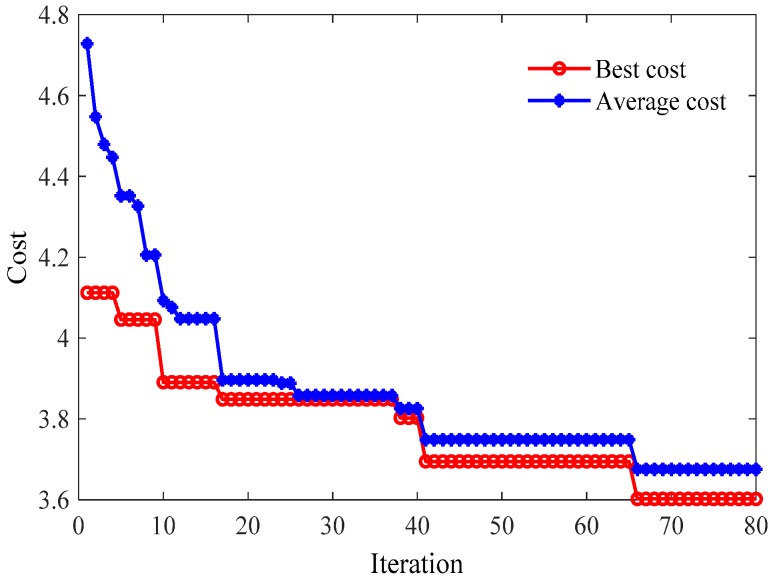
Convergence curves of ICA with Morlet W-LSSVR for C_2_H_4_ in Case 1.

Figure 2 shows the convergence process of ICA with Morlet W-LSSVR for C_2_H_4_. In Figure 2, the ordinate of the coordinates represents the cost of the empire, which is negatively correlated with fitness of the candidate solution, and the abscissa represents the iterations of ICA. It can be seen from Figure 2 that the both the “Best cost” and “Average cost” curves decrease during the iteration, which means the best fitness and the average fitness of empires are increased after iterations. In other words, the W-LSSVR can obtain more appropriate values for hyper-parameters as well as better performance after being optimized by ICA.

**Figure 3:**
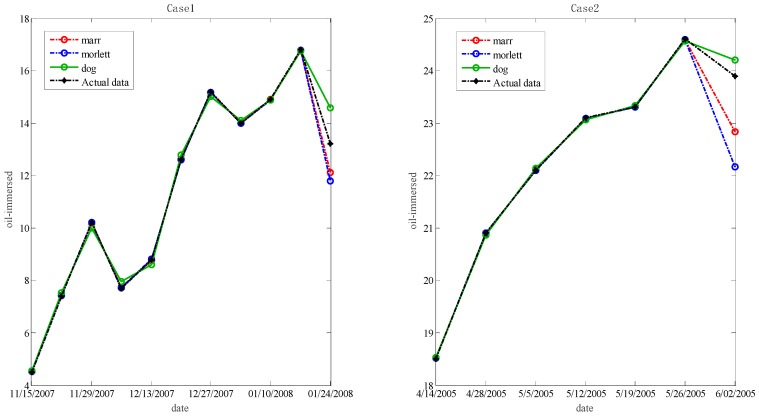
Forecasting results with three kinds of W-LSSVRs for C_2_H_6_ in Case 1 and Case 2.

**Figure 4:**
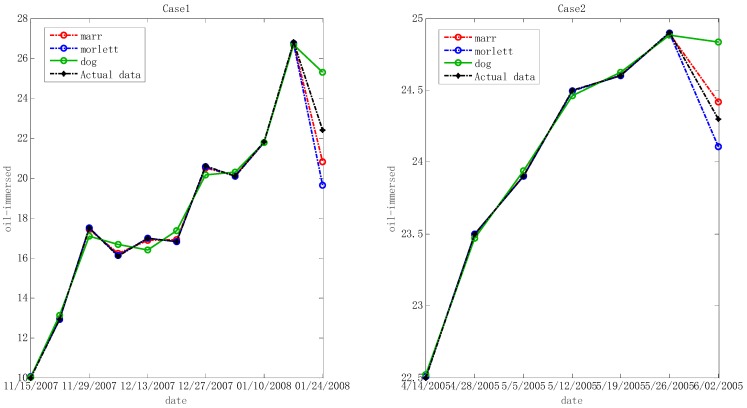
Forecasting results with three kinds of W-LSSVRs for C_2_H_4_ in Case 1 and Case 2.

**Figure 5:**
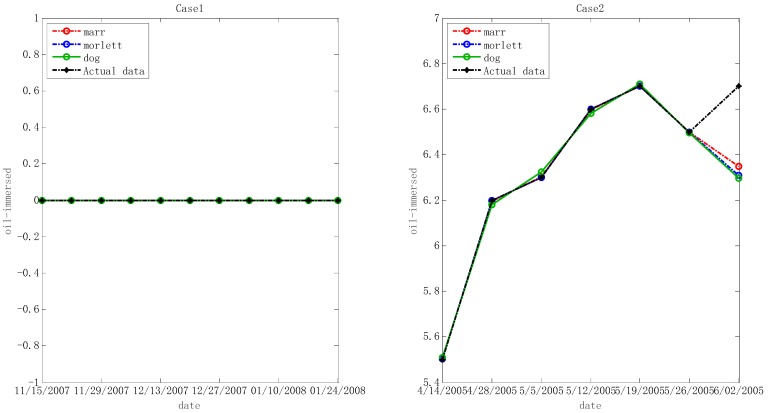
Forecasting results with three kinds of W-LSSVRs for C_2_H_2_ in Case 1 and Case 2.

**Figure 6:**
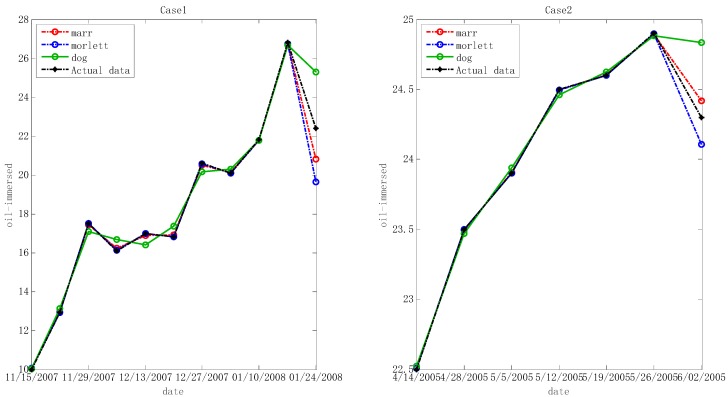
Forecasting results with three kinds of W-LSSVRs for CH_4_ in Case 1 and Case 2.

**Figure 7:**
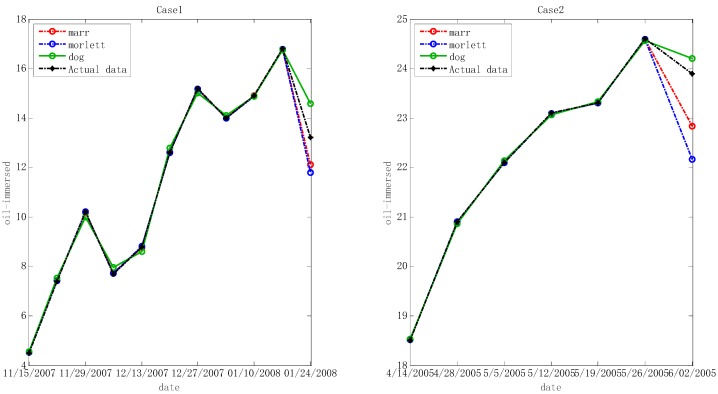
Forecasting results with three kinds of W-LSSVRs for H_2_ in Case 1 and Case 2.

**Figure 8:**
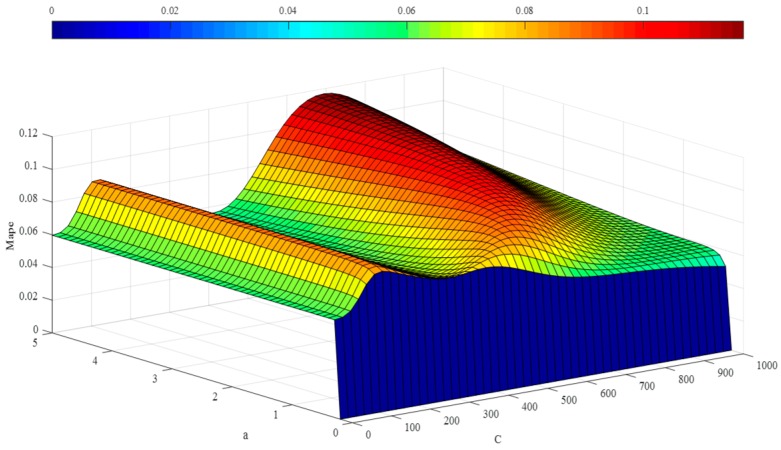
The relationship between *C*, *a*, and the MAPE of Morlet W-LSSVR for C_2_H_4_ in Case 1.

**Figure 9:**
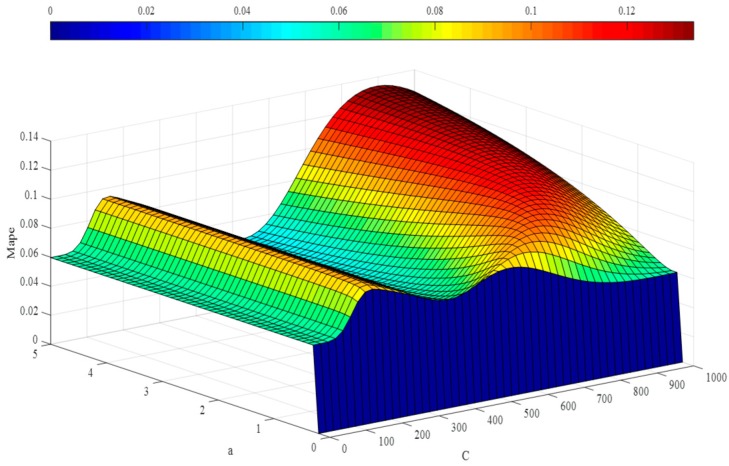
The relationship between *C*, *a*, and the MAPE of Marr W-LSSVR for C_2_H_4_ in Case 1.

**Figure 10:**
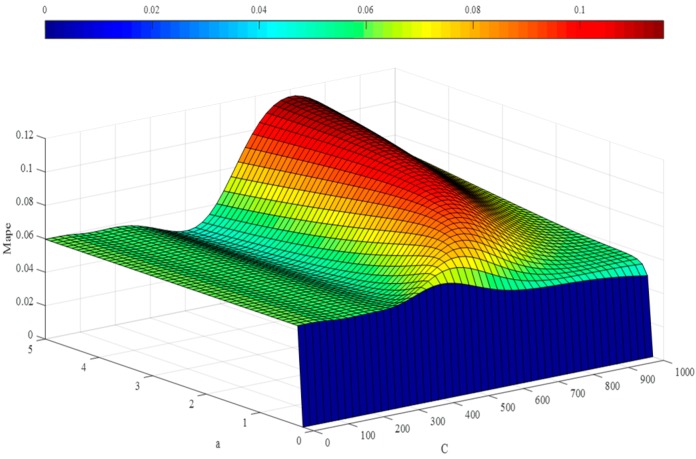
The relationship between *C*, *a*, and the MAPE of DOG W-LSSVR for C_2_H_4_ in Case 1.

In the step below, the optimal hyper-parameters are utilized to train Morlet, Marr and DOG W-LSSVR separately. The MAPE and *r*^2^ are proposed to measure the performance of the prediction model. The accuracy of the predicted outcome is examined by using the testing data. Morlet, Marr and DOG were respectively used as the wavelet kernel of W-LSSVR to predict in Case 1 and Case 2. The prediction results for the five gases are shown in the Figure 3, Figure 4, Figure 5, Figure 6 and Figure 7. It can be seen from Figure 3, Figure 4, Figure 5, Figure 6 and Figure 7 that the three kinds of W-LSSVR exhibit a favorable prediction performance as the prediction curves are almost identical to the curve of the actual value. Furthermore, the prediction curves of W-LSSVR with Morlet, Marr and DOG for five gases in Case 1 and Case 2 are almost coincident, therefore, the performance of W-LSSVR with any of the above three as the wavelet kernel is not very different. Figure 8, Figure 9 and Figure 10 illustrate the relationship between *C*, *a*, and the MAPE by using three kernel functions, respectively. The X-axis, Y-axis, and Z-axis represent *C*, *a* and MAPE, individually. Owing to the fact that Morlet, Marr and DOG W-LSSVR belong to the identical wavelet family and have some similar characteristics, the distinction between the performances is not that clear.

Table 1 shows the prediction performance and the optimal hyper-parameters of Morlet, Marr and DOG W-LSSVR. It can be seen from Table 1 that “Marr” wins the “top 1” seven times, followed by the “DOG” with two times. Furthermore, the “Marr” never ranked at the bottom, while the “Morlet” and “DOG” did so four and five times, respectively. According to the overall comparison of all gas prediction, the “Marr” is regarded as the most appropriate wavelet kennel for the W-LSSVR model and was adopted in our study.

### 5.2. Comparisons

Various forecasting models based on BPNN, SVR and PSO-W-LSSVR were performed in the same training and testing conditions for the purpose of comparison. It was necessary to normalize all the experimental data before training. Morlet W-LSSVR was chosen as an example to test and verify the forecasting accuracy. In the BPNN model, the most excellent network model for BPNN can be found by a hidden-layer network with the transfer function of log-sigmoid by training the BPNN 30 times to choose the best networks. Thus, the BPNN is generated by a hidden layer of 30 neurons and five input and output nodes. The training of BPNN takes the Levenberg Marquardt optimization method into account to minimize the expected default error value with the fastest speed.

**Figure 11:**
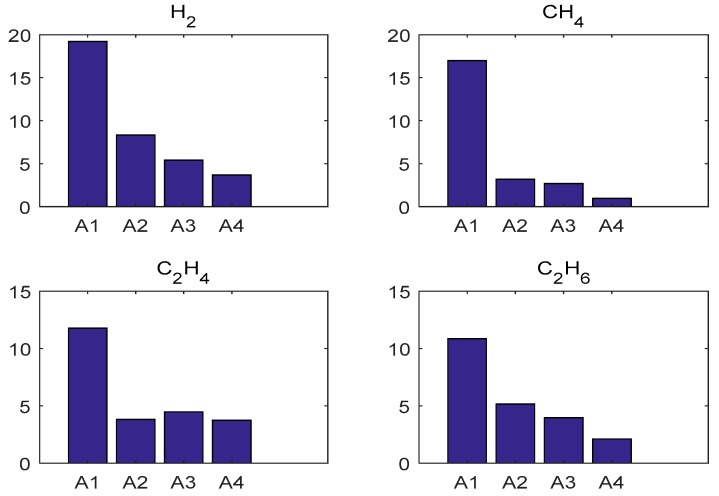
Testing MAPE results of the five forecasting approaches for gases in Case 1. (A1: BPNN, A2: SVR, A3: PSO-W-LSSVR, A4: ICA-W-LSSVR).

**Figure 12:**
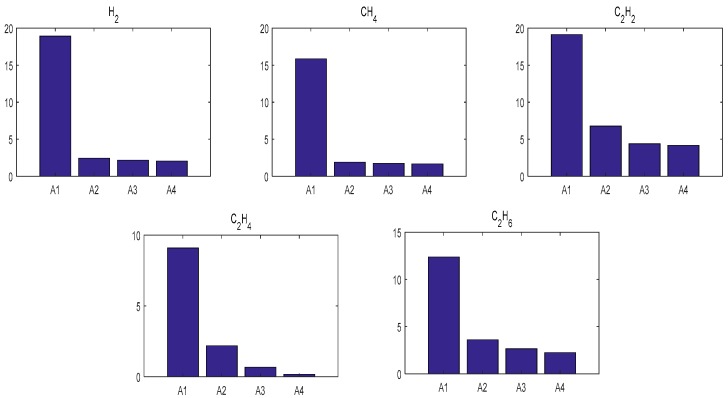
Testing MAPE results of the five forecasting approaches for gases in Case 2. (A1: BPNN, A2: SVR, A3: PSO-W-LSSVR, A4: ICA-W-LSSVR).

Table 2 illustrates the evaluation performances of BPNN, SVR, PSO-W-LSSVR and ICA-W-LSSVR in MAPE and *r*^2^. By analyzing Table 2, it can be seen that the learning ability of ICA-W-LSSVR during the training stage was excellent—its training error was less than 1% and its *r*^2^ close to 1. When comparing the data of the testing stage, it shows that the MAPE of ICA-W-LSSVR is less than 4%, which is much smaller than that of BPNN, SVR and PSO-W-LSSVR. The MAPE results for the four forecasting approaches for gases in the two Cases are shown in Figure 11 and Figure 12. As shown in Figure 11 and Figure 12, the W-LSSVR has a significantly better performance than the other three approaches. It can be inferred that W-LSSVR based on ICA has better prediction accuracy and generalization performance than the other methods.

## 6. Conclusions

In this paper, a novel method that can be used to predict dissolved gases in transformers by combining wavelet technology with LS-SVM is proposed. Test results showed that the proposed method is feasible—a high-precision prediction model for oil-dissolved gases in transformers has been established. The method effectively evaluates the transformer condition. Besides, the forecasting results were able to provide valuable information for the arrangement of maintenance schemes. There are several points to summarize this study, which are shown below:

1. In theory, arbitrary curves can be approximated in *L*_2_ (*R*^N^) space by the wavelet function that is known as a set of bases. Therefore, the wavelet technique is combined with LS-SVM to find a new forecasting method in this study. The results of the analysis infer that the admissible wavelet kernels, including Morlet, Marr and DOG wavelet kernels exist.
2. Simply, only two parameters need to be chosen in W-LSSVR as compared to the standard SVM regression. Moreover, the optimal hyper-parameters are available by applying the imperialist competition algorithm.
3. In many cases, the given forecasting procedure is effective to predict the useful gas contents in oil-dissolved transformers. The ICA based W-LSSVR has outstanding predicting ability for actual limited samples, and this is better than that of SVR, PSO-W-LSSVR and BPNN.

It is noted that this new method takes the fault diagnosis method into account to provide more useful information for future fault analysis of transformer polymer insulation. Therefore, a follow-up study needs to investigate this further.

## Figures and Tables

**Table 1:** The optimal parameters and prediction performance of Morlet, Marr and DOG W-LSSVR (◆: Morlet, ●: Marr, ■: DOG).

Case No.	Gas	Kernel	Hyper-Parameters		Testing	Ranking (1/2/3)
			*C*	*a*	MAPE (%)	
1	H_2_	Morlet	968.8347	10	4.0601	◇◇◆
		Marr	563.8497	6.7185	3.6785	●○○
		DOG	394.7711	6.4114	3.9803	□■□
	CH_4_	Morlet	822.7634	7.5923	1.1321	◇◆◇
		Marr	837.9421	1.4713	0.9675	●○○
		DOG	995.8278	2.0343	1.3872	□□■
	C_2_H_4_	Morlet	210.927	1.8828	3.9879	◇◆◇
		Marr	251.5736	2.8453	3.7385	●○○
		DOG	766.4636	7.8339	4.4210	□□■
	C_2_H_6_	Morlet	985.8278	6.5617	2.6413	◇◇◆
		Marr	961.6398	3.7656	2.1140	●○○
		DOG	995.8998	1.9911	2.3002	□■□
2	H_2_	Morlet	768.7547	8.9979	2.5740	◇◇◆
		Marr	833.8758	5.8930	2.0665	○●○
		DOG	394.7773	5.8830	1.8542	■□□
	CH_4_	Morlet	452.7316	7.8890	1.8003	◇◆◇
		Marr	797.8851	2.0754	1.6653	●○○
		DOG	989.4299	1.8997	1.9899	□□■
	C_2_H_2_	Morlet	517.8760	4.8877	4.3780	◇◆◇
		Marr	457.0041	8.5542	4.1681	●○○
		DOG	486.1128	9.0411	5.2119	□□■
	C_2_H_4_	Morlet	687.9904	2.0062	0.1949	◇◆◇
		Marr	774.8831	2.7831	0.1684	●○○
		DOG	882.1139	7.5572	0.2120	□□■
	C_2_H_6_	Morlet	456.1436	7.0032	2.4780	◇◇◆
		Marr	946.4432	3.6645	2.2409	○●○
		DOG	890.3323	2.3210	1.9930	■□□

**Table 2:** The evaluation performances of BPNN, RBFNN, GRNN and W-LSSVR in MAPE and *r*^2.^

Case No.	Gas	Training								Testing			
		MAPE (%)				*r* ^2^				MAPE (%)			
		BPNN	SVR	PSO-W-LSSVR	ICA-W-LSSVR	BPNN	SVR	PSO-W-LSSVR	ICA-W-LSSVR	BPNN	SVR	PSO-W-LSSVR	ICA-W-LSSVR
1	H_2_	14.9834	7.2456	0.1962	0.2446	0.7187	0.9168	0.9999	0.9999	19.2011	8.3248	5.4238	3.6785
	CH_4_	13.5612	2.6297	0.5499	0.5579	0.7479	0.968	0.9997	0.9995	16.98	3.198	2.6832	0.9675
	C_2_H_2_	/	/	/	/	/	/	/	/	/	/	/	/
	C_2_H_4_	9.9912	2.3216	0.3537	0.4932	0.8109	0.9999	0.9998	0.9995	11.7801	3.8227	4.4761	3.7385
	C_2_H_6_	10.5412	3.2884	0.1899	12.113	0.8322	0.9485	0.9999	0.8925	10.8611	5.1541	3.9606	2.114
2	H_2_	16.1476	1.1292	0.4872	1.2039	0.7632	0.976	0.9999	0.9787	18.9187	2.4628	2.1567	2.0665
	CH_4_	14.0121	0.8325	0.3071	0.3112	0.81	0.9815	0.9999	0.9859	15.8601	1.9107	1.7543	1.6653
	C_2_H_2_	18.1890	3.4673	0.5194	0.8878	0.7511	0.971	0.9998	0.9178	19.1222	6.7795	4.385	4.1681
	C_2_H_4_	8.9832	0.5231	0.0871	0.97	0.8532	0.9785	0.9999	0.9303	9.1011	2.1942	0.6741	0.1684
	C_2_H_6_	11.9867	1.3453	0.5184	0.4366	0.7765	0.9626	0.9998	0.9622	12.3901	3.609	2.6521	2.2409
